# Pituitary spindle cell oncocytoma: Two cases report and literature review

**DOI:** 10.1016/j.ijscr.2024.110328

**Published:** 2024-09-21

**Authors:** Yi-Ying Hsieh, Shuo-Chi Chien, Hong-Chieh Tsai, Kuo-Chen Wei, Chi-Cheng Chuang, Shih-Ming Jung

**Affiliations:** a Department of Neurosurgery, Chang Gung Memorial Hospital, Linkou Center, Taoyuan City, Taiwan; b Department of Pathology, Chang Gung Memorial Hospital, Linkou Center, Taoyuan City, Taiwan

**Keywords:** Pituitary spindle cell oncocytoma, TTF-1, Gamma knife

## Abstract

**Introduction:**

Pituitary spindle cell oncocytoma (PSCO) is a seldom-encountered type of pituitary neoplasm with distinctive histological features. It was first described as a distinct entity by Roncaroli et al. in 2002. We present two cases of PSCO and discuss its clinical, radiological, and histopathological features, along with a review of the existing literature.

**Presentation of case:**

Two cases underwent trans-nasal transsphenoidal surgery for tumor resection and had different treatment following would be discussed in this article. Both had unique pathology pattern as Pituitary spindle cell oncocytoma.

**Discussion:**

Tumors positive for TTF-1 in the sellar region, such as pituicytoma, granular cell tumor, and spindle cell oncocytoma, originate from the posterior pituitary gland and are rare. The expression of thyroid transcription factor-1 (TTF-1) in these tumors aids in distinguishing them from other pituitary neoplasms.

**Conclusion:**

Pituitary spindle cell oncocytoma is a rare entity among pituitary tumors. This case report highlights the clinical, radiological, histopathological, and immunohistochemical features of PSCO. Surgeons and pathologists should consider this rare diagnosis in patients with sellar and suprasellar masses, as early recognition and complete surgical resection can lead to favorable outcomes.

## Highlights

• Pituitary spindle cell oncocytoma (PSCO) is a seldom-encountered type of pituitary neoplasm with distinctive histological features.
• Tumors positive for TTF-1 in the sellar region, such as pituicytoma, granular cell tumor, and spindle cell oncocytoma, originate from the posterior pituitary gland and are rare.
• PSCO is recognized as a highly vascular tumor, posing significant challenges during surgical resection due to the risk of considerable intraoperative bleeding.

## 1 Introduction

Pituitary spindle cell oncocytoma (PSCO) is a seldom-encountered type of pituitary neoplasm with distinctive histological features. It was first described as a distinct entity by Roncaroli et al. in 2002 [1]. Since then, only a limited number of cases have been reported in the literature. PSCO typically presents with non-specific clinical symptoms and is often discovered incidentally on radiological imaging. We present two cases of PSCO and discuss its clinical, radiological, and histopathological features, along with a review of the existing literature.

## 2 Methods

Two cases were reviewed in our hospital to identify the specific pathological trait of pituitary spindle cell oncocytoma. The study is retrospective and both of the patient receive trans-nasal transsphenoidal surgery for tumor resection. The work has been reported in line with the SCARE criteria [2].

## 3 Case 1 report

**Fig. 1:**
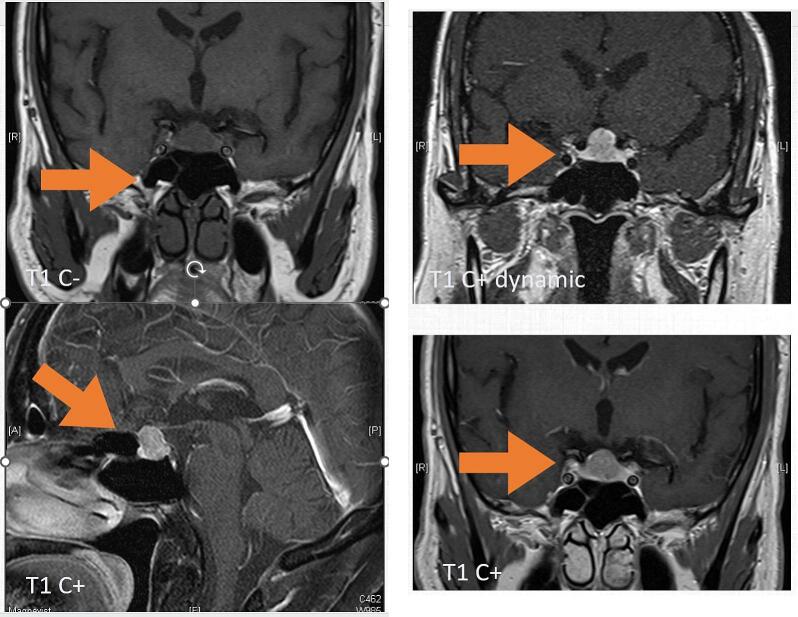
Pre-operative MRI of Case 1.

A 69-year-old male patient with past medical history of hypertension, mitral regurgitation and benign prostate hyperplasia presented to our neurology clinic with right facial pain for 4 years. He denied headache, blurred vision, vision defect and limbs weakness. There is no focal visual deficit but mild visual acuity was complained. Brain MRI revealed sellar and suprasellar lesion, suspect pituitary tumor. Then, he was referred to neurosurgical clinic. MRI of Sella Turcica with enhancement showed a favored posterior pituitary gland tumor up 1.4 cm in height to with suprasellar extension and causing optic chiasm compression, suspect pituitary gland macroadenoma (Fig. 1). Endocrine assessment showed no hormonal abnormalities.

**Fig. 2:**
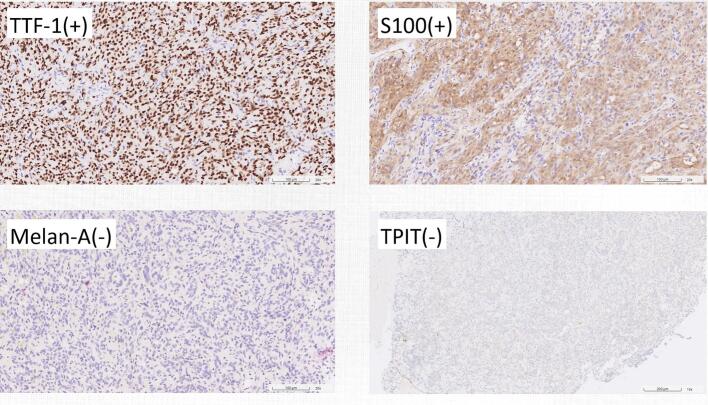
Immunohistochemistry study of Case 1.

The patient underwent endoscopic trans-nasal transsphenoidal surgery for tumor resection. Gross examination of the surgical specimen revealed a tan-pink, soft, and well-circumscribed mass. Microscopic examination showed interlacing fascicles and poorly defined lobules of spindle to epithelioid cells with eosinophilic, variably granular cytoplasm and some pigments. Immunohistochemistry demonstrated strong positivity for S-100 protein, EMA(GP1.4) and TTF-1, while being negative for neuroendocrine markers such as synaptophysin and chromogranin (Fig. 2). The histopathological findings were consistent with a diagnosis of pituitary spindle cell oncocytoma.

Follow-up MRI demonstrated pituitary spindle cell oncocytoma status post-surgical resection with residual tumor at suprasellar region (0.8 × 0.7 × 0.4 cm) and left pituitary fossa (0.5 × 0.5 × 0.6 cm). He remained asymptomatic and discharged smoothly.

## 4 Case 2 report

A 68-year-old female had gradually loss her visual accuracy for few months. Visual field examination showed bi-temporal visual defect especially. Several survey was done and CT of brain showed a 3.5 cm × 2.5 cm × 3.1 cm huge sellar mass with mass effect over brainstem and optic chiasm likely pituitary macroadenoma and a 2 cm meningioma in right frontal base. *Trans*-Sphenoid approach for subtotal removal of sellar mass was smoothly done and post-operative MRI showed 2 × 1.5 cm residual tumor. Immunohistochemical study of pathology report of this patient revealed strong positivity for S-100 protein, EMA(E29) and TTF-1. Stereotactic radiosurgery (500 × 5 cGy) was arranged for residual tumor control. 2 years follow-up MRI showed partial size decreased of the tumor.

## 5 Discussion

Pituitary spindle cell oncocytoma (PSCO) is an exceptionally rare type of pituitary tumor. Clinically, it often presents similarly to other pituitary adenomas, potentially delaying diagnosis. Radiologically, PSCO can resemble other sellar and suprasellar lesions [3]. The definitive diagnosis of PSCO is achieved through histopathological examination, which reveals characteristic spindle-shaped cells with oncocytic features. Immunohistochemistry is essential for confirmation, typically showing positivity for TTF-1, S-100 protein, vimentin, and cytokeratin AE1/AE3 [4].

Since Pituitary spindle cell oncocytoma is a highly vascular tumors that can cause significant bleeding intra-operatively. One study showed pre-operative embolization at second operation for pituitary spindle cell oncocytoma showed still significant intraoperative bleeding even if embolization is done [5]. Feeders chosen for embolization is also limited due to antegrade blood flow should be preserved. Preoperative embolization was not done at our two cases due to unknown pathology before operation. Besides tumor bleeding, anatomy of cavernous sinus and ICAs would help us prevent vessel injury during the operation. Over-packing for control bleeding may result in the compression of cranial nerves and even loss of the blood flow nearby [6]. Thus, adequate packing using hemostatic matrix as such as floseal or two-surgeon operation would help for bleeding control.

Tumors positive for TTF-1 in the sellar region, such as pituicytoma, granular cell tumor, and spindle cell oncocytoma, originate from the posterior pituitary gland and are rare. The expression of thyroid transcription factor-1 (TTF-1) in these tumors aids in distinguishing them from other pituitary neoplasms [7]. Pituicytoma is a benign tumor that generally presents as an intrasellar mass, causing visual disturbances and pituitary dysfunction. Granular cell tumors, composed of cells with abundant granular cytoplasm, typically occur in adults and have low malignant potential. Spindle cell oncocytoma is defined by spindle-shaped cells with oncocytic cytoplasm. Although these tumors exhibit diverse radiological features, TTF-1 positivity in immunohistochemical staining is crucial for diagnosis. Surgical resection is usually the preferred treatment for these tumors.

PSCO is recognized as a highly vascular tumor, posing significant challenges during surgical resection due to the risk of considerable intraoperative bleeding. Preoperative embolization is vital in reducing the tumor's vascularity, thereby minimizing intraoperative blood loss and facilitating safer resection. When complete resection is not feasible, adjuvant radiotherapy is recommended to manage the residual tumor. Gamma knife radiosurgery, in particular, has shown efficacy in controlling tumor growth and preventing recurrence. The prognosis for patients with PSCO largely depends on the extent of surgical resection and the effectiveness of any adjuvant therapies used [8]. Patients generally have a favorable prognosis if complete resection is achieved, with adjuvant radiotherapy providing good local control in cases of residual disease. The most important document for tumor recurrence is the level of surgical excision [8].

## 6 Conclusion

Pituitary spindle cell oncocytoma is a rare entity among pituitary tumors. This case report highlights the clinical, radiological, histopathological, and immunohistochemical features of PSCO. Surgeons and pathologists should consider this rare diagnosis in patients with sellar and suprasellar masses, as early recognition and complete surgical resection can lead to favorable outcomes.

## Consent

Written informed consent was obtained from the patients for publication and any accompanying images. A copy of the written consent is available for review by the Editor-in-Chief of this journal on request.

## Ethical approval

Ethical approval for this study (IRB number 202401058B0) was provided by the Institutional Review Board of Chang Gung Memorial Hospital, linkou, Taiwan on 1 July 2024.

## Funding

N/A.

## Author contribution

Yi-Ying Hsieh: Conceptualization, Investigation, Writing - Original Draft, Review & Editing; Kuo-Chen Wei: Validation, Supervision, Project administration, Writing - Review & Editing; Hong-Chieh Tsai: Validation, Data Curation, Writing - Review & Editing; Chi-Cheng Chuang: Validation, Resources, Writing - Review & Editing; Po-Hung Chang: Methodology, Resources, Writing - Review & Editing; Shih-Ming Jung: Methodology, Resources, Writing - Review & Editing Shuo-Chi Chien: Investigation, Resources, Writing - Original Draft, Review & Editing, Supervision, Project administration.

## Guarantor

Shuo-Chi Chien

Department of Neurosurgery, Chang Gung Memorial Hospital, Linkou.

No. 5, Fu-Xin St., Taoyuan, 333, Taiwan

Tel: (886) 3-328-1200 extension 2412

Email: mp1886@cgmh.org.tw

## Conflict of interest statement

N/A.
